# Fly-Tox: A panel of transgenic flies expressing pest and pollinator cytochrome P450s

**DOI:** 10.1016/j.pestbp.2020.104674

**Published:** 2020-10

**Authors:** Amy McLeman, Bartlomiej J. Troczka, Rafael A. Homem, Ana Duarte, Christoph Zimmer, William T. Garrood, Adam Pym, Katherine Beadle, Rebecca J. Reid, Vassilis Douris, John Vontas, T.G. Emyr Davies, Richard ffrench Constant, Ralf Nauen, Chris Bass

**Affiliations:** a College of Life and Environmental Sciences, Biosciences, University of Exeter, Penryn Campus, Penryn, Cornwall, UK; b Department of Biointeractions and Crop Protection, Rothamsted Research, Harpenden, UK; c Institute of Molecular Biology & Biotechnology, Foundation for Research & Technology Hellas, Crete, Greece; d Department of Biological Applications and Technology, University of Ioannina,45110 Ioannina, Greece; e Department of Crop Science, Agricultural University of Athens, Athens, Greece; f Bayer AG, Crop Science Division, R&D, Alfred Nobel-Strasse 50, 40789.Monheim, Germany

**Keywords:** Drosophila, Transgenic, Cytochrome P450, Toxicology, Insecticide

## Abstract

There is an on-going need to develop new insecticides that are not compromised by resistance and that have improved environmental profiles. However, the cost of developing novel compounds has increased significantly over the last two decades. This is in part due to increased regulatory requirements, including the need to screen both pest and pollinator insect species to ensure that pre-existing resistance will not hamper the efficacy of a new insecticide via cross-resistance, or adversely affect non-target insect species. To add to this problem the collection and maintenance of toxicologically relevant pest and pollinator species and strains is costly and often difficult. Here we present Fly-Tox, a panel of publicly available transgenic *Drosophila melanogaster* lines each containing one or more pest or pollinator P450 genes that have been previously shown to metabolise insecticides. We describe the range of ways these tools can be used, including in predictive screens to avoid pre-existing cross-resistance, to identify potential resistance-breaking inhibitors, in the initial assessment of potential insecticide toxicity to bee pollinators, and identifying harmful pesticide-pesticide interactions.

## Highlights

• *Drosophila melanogaster* is a powerful model system to study cytochrome P450-mediated insecticide tolerance.
• Fly-tox comprises a panel of transgenic *D. melanogaster* lines expressing P450s from insect pest and pollinator species.
• Fly-tox can be used in numerous ways to characterise the role of pest/pollinator P450s in insecticide metabolism.
• We describe the applications of Fly-Tox and provide detailed protocols on its use.
• The benefits and limitations of the system are discussed.

## Graphical abstract

**Figure:**
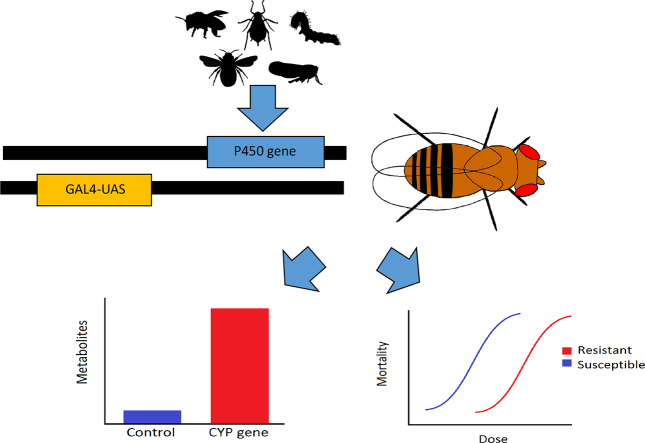

## 1 Introduction

The development of novel insecticides has never been more challenging. There are a number of reasons for this, (see (Sparks, 2013) for an excellent review of this topic), including the requirement to develop a product that is unaffected by pre-existing resistance and is highly specific for the target pests. Consequently, a key component of the insecticide discovery process involves screening a range of insect pest strains, which exhibit resistance to one or more existing insecticide, and key non-target species - including certain bee pollinators. The maintenance and testing of such species is costly, difficult and in some cases only possible at certain times of the year (i.e. in the case of certain bee species). There is thus an urgent need to develop tools that may be used year round in early screens to support the discovery of novel lead compounds.

Insects have evolved sophisticated systems to metabolise the natural xenobiotics they encounter in their environment (Li et al., 2007) and these can be critical determinants of their sensitivity to synthetic insecticides, and/or co-opted during the evolution of resistance (Dermauw et al., 2018; Manjon et al., 2018). One of the most important of these biotransformation systems are cytochrome P450 monooxygenases (P450s), a superfamily of enzymes which are ubiquitous in nature and metabolise a remarkable array of exogenous and endogenous compounds (Schuler, 1996). Insect P450s have been shown to mediate resistance to a wide range of different pesticide classes in numerous arthropod pests (Scott, 1999). More recent work has demonstrated that P450s can also be key determinants of the sensitivity of bee species to pesticides and can provide strong intrinsic tolerance to certain compounds (Manjon et al., 2018). Below we provide a brief background on the P450s included in the Fly-Tox panel and direct the reader to the appropriate citation(s) for further details.

### 1.1 *Nilaparvata lugens* - CYP6ER1, CYP6AY1

Two P450s have been shown to confer resistance to neonicotinoids in the brown planthopper, *N. lugens*, an important pest of rice throughout Asia (Kiritani, 1979). The first of these, CYP6ER1, is consistently overexpressed in resistant strains of *N. lugens* throughout Asia (Garrood et al., 2016; Bass et al., 2011a). A range of functional approaches including RNAi (Pang et al., 2016) and in vitro and in vivo functional expression has demonstrated that this P450 confers resistance to imidacloprid, thiamethoxam, dinotefuran and clothianidin (Bao et al., 2016). More recent work has demonstrated that marked genetic variation in the coding sequence of this P450 is observed in populations of brown planthopper collected from across Asia, but just two sequence variants (CYP6ER1vA and CYP6ER1vB) are highly overexpressed in resistant strains and metabolise imidacloprid (Zimmer et al., 2018). CYP6AY1 was initially identified as overexpressed in neonicotinoid resistant populations of *N. lugens* from China (Bass et al., 2011b; Ding et al., 2013). The capacity of this P450 to metabolise imidacloprid, and confer resistance, has been clearly demonstrated using a range of functional approaches (Bao et al., 2016; Ding et al., 2013). However, recent extensive monitoring programs have shown that, in contrast to *CYP6ER1*, this P450 is not overexpressed but rather down-regulated in most of the neonicotinoid populations sampled across Asia, including those from China (Garrood et al., 2016; Wu et al., 2018). Thus these findings suggest *CYP6AY1* is of less relevance to resistance in the field than *CYP6ER1*.

### 1.2 *Myzus persicae* - CYP6CY3, CYP6CY4

The peach potato aphid *Myzus persicae* is a highly polyphagous global crop pest (Van Emden et al., 1969). The P450 CYP6CY3 is overexpressed in the tobacco-adapted subspecies of this aphid (*Myzus persicae nicotianae*) which exhibits resistance to both the natural insecticide nicotine and synthetic neonicotinoids (Bass et al., 2014). In vivo and in vitro functional expression has demonstrated that this P450 efficiently detoxifies nicotine and the neonicotinoids, imidacloprid and clothianidin (Bass et al., 2013; Nakao et al., 2019). Recent work has also suggested that this resistance mechanism has spread to populations of *M. persicae* on other host plants, likely as a result of the fitness benefits it provides in the presence of neonicotinoid insecticides (Bass et al., 2013). Our recent work has shown that *CYP6CY3* is amplified as part of a large amplicon in resistant clones. The amplified region includes a second P450, *CYP6CY4*, which also has the capacity to break down nicotine, and likely neonicotinoids (Singh et al., 2020).

### 1.3 *Plutella xylostella* - CYP6BG1

CYP6BG1 was first linked to resistance to the pyrethroid permethrin in diamondback moth, *Plutella xylostella*, an economically important pest of cruciferous crops worldwide (Bautista et al., 2007). RNAi was subsequently used to provide functional evidence that this P450 contributes to resistance (Bautista et al., 2009). More recently, however, CYP6BG1 has been linked to resistance to the diamide insecticide chlorantraniliprole. In two independent studies *CYP6BG1* was found to be overexpressed in diamide resistant *P. xylostella* populations (Li et al., 2018; Lin et al., 2013). Both RNAi knockdown and transgenic expression in *D. melanogaster* have provided support for a causal role of this P450 in resistance (Li et al., 2018). However, in a following study, transgenic expression in Drosophila failed to confer resistance to chlorantraniliprole, with transgenic flies significantly more sensitive to this compound than a control line lacking the transgene (Mallott et al., 2019). One possible explanation for this discrepancy may be differences in the bioassay methodology employed in the two studies with the former using a larval assay and the latter testing adults.

### 1.4 *Bemisia tabaci* - CYP6CM1

*Bemisia tabaci*, the tobacco whitefly, is a major sucking pest of over 900 host plant species encompassing fruit, vegetables and ornamentals (Sparks and Nauen, 2015). The *B. tabaci* P450 CYP6CM1 was originally shown to confer resistance to the neonicotinoid insecticide imidacloprid (Karunker et al., 2008). Further functional analysis has revealed that it also detoxifies the neonicotinoids clothianidin and thiacloprid, although not acetamiprid or thiamethoxam (Roditakis et al., 2011). Interestingly, overexpression of this enzyme has also been shown to confer cross-resistance to the transient receptor potential **cation channel** subfamily V (TRPV) cation channel blocker pymetrozine, a chemically unrelated insecticide with a completely different mode of action (Nauen et al., 2013).

### 1.5 *Brassicogethes (syn. Meligethes) aeneus –* CYP6BQ23

The pollen beetle, *Meligethes aeneus*, is a major coleopteran pest of oilseed rape (*Brassica napus*) throughout much of Europe (Free and Williams, 1979). Resistance to pyrethroid insecticides was linked to the marked (up to 900-fold) overexpression of this P450 in adults and larvae of pyrethroid resistant strains (Zimmer et al., 2014). Recombinant expression of CYP6BQ23 in an insect cell line showed that it can hydroxylate deltamethrin and *tau*-fluvalinate demonstrating its causal role in resistance (Zimmer et al., 2014).

### 1.6 *Tribolium castaneum* – CYP6BQ9

The red flour beetle, *Tribolium castaneum*, is a worldwide pest of stored grains (Sokoloff, 1977). Resistance to deltamethrin in this species has been linked to overexpression of the P450 *CYP6BQ9* in the insect brain (Zhu et al., 2010). RNAi and in vitro expression has proved that this P450 metabolises deltamethrin to 4-hydroxy deltamethrin leading to resistance (Zhu et al., 2010). Furthermore, transgenic expression of *CYP6BQ9* in the brain of *D. melanogaster* conferred a resistance phenotype, providing one of the first examples of using Drosophila to validate candidate P450 genes of agricultural pests (Zhu et al., 2010).

### 1.7 *Helicoverpa armigera* – CYP337B3

The cotton bollworm, *Helicoverpa armigera*, is a polyphagous pest causing damage to a range of cereal and vegetable crops (Ahmad, 2007). A chimeric cytochrome P450 gene, *CYP337B3*, is thought to have arisen by unequal crossing-over between the P450 genes, *CYP337B1* and *CYP337B2* (Joußen et al., *2012*). This has been identified as a key mechanism of resistance to the pyrethroids fenvalerate and cypermethrin in *H. armigera* populations worldwide (Joußen et al., 2012; Rasool et al., 2014). The capacity of this P450 to metabolise fenvalerate has been demonstrated using heterologous expression (Joußen et al., 2012).

### 1.8 *Apis mellifera*, *Bombus terrestris*, *Osmia bicornis* – CYP9Q1–6, CYP9BU1–2

Recent research has shown that several managed bee species have specific P450 enzymes that are preadapted to confer intrinsic tolerance to some insecticides. P450s within the CYP9Q subfamily in honey bees, *Apis mellifera*, bumblebees, *Bombus terrestris* (Manjon et al., 2018), and a closely related subfamily (CYP9BU) in the red mason bee, *Osmia bicornis* (Beadle et al., 2019) were found to metabolise the *N*-cyanoamidine neonicotinoids thiacloprid and acetamiprid much more efficiently than the *N*-nitroguanidine compound imidacloprid. Of these P450s, *CYP9Q3* (*A. mellifera*), *CYP9Q4* and *CYP9Q6* (*B. terrestris*) and *CYP9BU1* (*O. bicornis*) conferred resistance in transgenic flies (Manjon et al., 2018; Beadle et al., 2019). Previous work has also demonstrated that recombinant *CYP9Q1–3* can metabolise the pyrethroid *tau*-fluvalinate and the organophosphate coumaphos, two insecticides that show marked selectivity for mites (i.e., Varroa) over bees (Mao et al., 2011). Thus, these appear to be important generalist P450s and key determinants of the sensitivity of these bee species to many insecticides.

In contrast to the difficulty of rearing and testing many pest and pollinator species, bioassays of the model insect *D. melanogaster* are simple, inexpensive and rapid to perform. Transformation of *D. melanogaster* is also routine and site-specific integration using strains containing docking sites, in combination with an integrase such as phiC31, allows genes to be integrated at specific loci in the genome (Bateman and Lee, 2006). This targeted approach has the additional advantage that multiple lines generated in the same way can be compared, as the positional effects intrinsic to random integration can be avoided. Another resource available in *D. melanogaster* is the GAL4/UAS system which can be used to dictate when and where a transgene is expressed (Brand and Perrimon, 1993). This binary system utilises a fly strain expressing the yeast transcription factor GAL4 which, when crossed to a second strain containing a gene of interest, inserted next to an upstream activation sequence (UAS), activates its expression. This results in the transgene being transcribed in the F1 progeny of this cross, based on the expression pattern of GAL4 in the driver line. A huge number of fly lines are available to the scientific community which express GAL4 under a range of different promoters that differ in their spatiotemporal expression pattern (McGuire et al., 2004). These tools have been widely used to study *D. melanogaster* genes of interest, however, more recently they have also been used to study the function of P450s from pest and pollinator insect species (Manjon et al., 2018; Beadle et al., 2019; Pym et al., 2019; Daborn et al., 2007; Scott and Buchon, 2019; Homem and Davies, 2018; Daborn et al., 2012).

Here we present Fly-Tox, a panel of publicly available transgenic *D. melanogaster* lines, each containing one or more pest or pollinator P450 genes which have been previously shown to metabolise one or more insecticide, and describe the range of ways these tools can be used.

## 2 Methods

### 2.1 Creation of transgenic fly lines

All genes described in this study were codon optimised for *D. melanogaster* expression and synthesised except *CYP9Q6*. Gene accession numbers are provided in Table 1. All constructs were cloned into the *pUASTattB* plasmid (GenBank: EF362409.1) and purified using the QIAprep Spin Miniprep Kit (Qiagen), with DNA eluted using water (as the elution buffer provided with this kit is toxic to flies).

**Table 1:** Summary of the transgenic lines included in the Fly-Tox panel.

Single transgene	Gene accession number	Species	BDSC stock number	Compounds tested	Resistance ratio*	Fly line reference	Notes
CYP6CY3	HM009309	*Myzus persicae*		Nicotine	5.7	Bass et al., 2013 (Bass et al., 2013)	Lines generated by C. Bass lab
CYP6CY4	NP_001352549				3.7	Singh et al., 2020	
CYP6ER1vL	MF970461	*Nilaparvata lugens*		Imidacloprid	0.4	Unpublished	Lines generated by Rothamsted Research and C. Bass lab
CYP6ER1vA	MF970458				4		
CYP6ER1vB	MF970459				3		
CYP6ER1vC	MF970460				1.5		
CYP6ER1_P377del	See CYP6ER1vL				4.5	Zimmer et al., 2018 (Zimmer et al., 2018)	Lines generated by Rothamsted Research and C. Bass lab
CYP6ER1_A375del + A376G					20		
CYP6ER1_T318S + P377del					20		
CYP6ER1_T318S + A375del + A376G					35		
CYP6ER1_T318S					20		
CYP6AY1	AJ852423				1.7	Unpublished	CYP link to resistance published in Wu et al., 2018 (Wu et al., 2018) Line in this study generated by C. Bass lab
CYP6BG1	KX844829	*Plutella xylostella*		Chlorantranili-prole	0.3	Mallott et al., 2019 (Mallott et al., 2019)	Line generated by C. Bass lab
CYP6CM1	GQ214539	*Bemisia tabaci*		Imidacloprid	0.9	Daborn et al., 2012 (Daborn et al., 2012)	Confers resistance in published study (larval assay) Lines in this study generated by C. Bass lab
CYP6BQ23	KC840055	*Meligethes aeneus*		Deltamethrin	0.7	Unpublished	Lines in this study generated by C. Bass lab
CYP6BQ9	GU727868	*Tribolium castaneum*			1.3	Zhu et al., 2010 (Zhu et al., 2010)	
CYP337B3	JQ284029	*Helicoverpa armigera*		Fenvalerate	0.6	Unpublished	CYP link to resistance published in Joußen et al., 2012 (Joußen et al., 2012). Line in this study generated by C. Bass lab
CYP9Q1	XP_006562364	*Apis mellifera*		Thiacloprid	2.7	Manjon et al., 2018 (Manjon et al., 2018)	Heat shock drivers used in the study. These lines have also been tested against acetamiprid (see original publication). Lines generated by Rothamsted Research
CYP9Q2	XP_392000				3.4		
CYP9Q3	XP_006562363				7.4		
CYP9Q4	XP_003393377	*Bombus terrestris*			4.9		
CYP9Q5	XP_003393376				3.4		
CYP9Q6	MK559424				3.9	Troczka et al., 2019 (Troczka et al., 2019)	Line generated by Rothamsted Research.
CYP9BU1	MH500604	*Osmia bicornis*		Thiacloprid	3.8	Beadle et al., 2019 (Beadle et al., 2019)	These lines have also been tested against imidacloprid (see original publication)
CYP9BU2	MH500605				1.1		
CYP9R38	MH500606				0.6		
	
Combinations							
CYP9Q2; CYP9Q3	See CYP9Q1–3	*Apis mellifera*		Thiacloprid	10.3	Unpublished	All lines generated by Rothamsted Research
CYP9Q1/CYP9Q3					4.3		
CYP9Q1/CYP9Q2; CYP9Q3					9.8		CYP9Q3 is on the 3rd chromosome
CYP9Q1–3;+					2.9		All 3 genes present on a single plasmid

Constructs (200 ng/μl) were injected into preblastodermal embryos of transgenic *D. melanogaster* strains carrying an *attP* docking site and the *phiC31* integrase gene under the control of the *vasa* regulatory region. For detailed genotypes of the strains see Table S1. Injected eggs were placed in optimal conditions and allowed to develop to adulthood. Adults were collected before reaching sexual maturity and crossed with flies of the same strains. The expression of the *mini-white* gene, which is present in the *pUASTattB* plasmid and acts as an eye colour marker, was used to select for positive integration of the transgenes in the genome of F1 flies. Positive F1 flies were selected and inter-crossed to generate homozygous individuals which were selected based on the intensity of their eye colour. Whilst heterozygous presented yellow eyes, homozygous counterparts showed a dark orange eye phenotype. These were selected and inter-crossed to generate homozygous lines with PCR and sequencing further confirming transgene integrations. Genomic DNA was extracted from pools of 10 flies for each stock using the Plant DNeasy Mini kit (Qiagen) following the manufacturers protocol. 20 ng of this DNA was used as template in PCR using Phusion DNA polymerase (Thermo) following the manufacturers protocol and the primers listed in Table S2 (supplementary material 1). Thermocycling conditions consisted of an initial denaturation step at 98 °C for 30 s, followed by 35 cycles of 98 °C for 10 s, 55 °C for 20 s, 72 °C for 1 min, and a final extension at 72 °C for 5 min. Products were direct Sanger sequenced using the primers detailed in Table S2. All flies were reared on NutriFly food (NLS) at 24 °C, 60% RH and 12 h light/dark cycles.

### 2.2 Driving the expression of the P450s in Drosophila

The GAL4/UAS system was used to regulate the expression of P450 transgenes in Drosophila; for a detailed crossing scheme refer to Supplementary methods file 1. Briefly, this system is made of two components, the yeast transcription factor GAL4 and its enhancer region, an upstream activating sequence (UAS). GAL4 expression can be regulated by Drosophila endogenous promoters and thousands of strains have already been generated and characterised, these are readily available from the Bloomington Drosophila Stock Center (BDSC https://bdsc.indiana.edu/index.html). To regulate the expression of a gene of interest, one must first generate a transgenic strain carrying such a gene in frame with the UAS (as described above). This strain can then be crossed to a suitable GAL4 strain to induce the expression of the transgene in the progeny of this cross in a spatiotemporal controlled manner. For a list of all GAL4 strains used to drive the expression of CYP transgenes see Table S1.

To confirm transgene expression, qPCRs were performed on the F1 flies. Total RNA was extracted from 4 pools of 10 adult flies of each line using the ISOLATE II RNA Mini Kit (Bioline) and reverse transcribed to cDNA using Superscript III reverse transcriptase (Invitrogen) and random hexamers (Invitrogen) following manufacturer protocols in both cases. PCR reactions (20 μl) contained 10 ng of cDNA, 10 μl of SYBR Green JumpStart Taq Readymix (Sigma), and 0.25 μm of each primer. Samples were run on a Rotor-Gene 6000 (Corbett Research) using temperature cycling conditions of: 2 min at 95 °C followed by 40 cycles of 95 °C for 15 s, 57 °C for 15 s and 72 °C for 20 s. Data were analysed in Microsoft Excel according to the ΔΔC_T_ method (Rao et al., 2013) using the *RPL32* reference gene for normalization (Ponton et al., 2011). Full dose response bioassays were performed by feeding adult female flies (~5 days post eclosion) a range of insecticide concentrations dissolved in sugar/agar at room temperature (~22 °C) with 12:12 h light:dark cycle. At least three replicates of 20 flies were carried out for each concentration. LC_50_ values were calculated using probit analysis in Genstat (Payne, 2009) (VSN International).

### 2.3 Microsomal preparations from flies expressing bee P450s and insecticide metabolism assays

Flies for insecticide metabolite studies were generated as described above using a heat-shock inducible GAL4 strain (Table S1). Transgene expression was induced by a 30 min heat shock treatment at 37 °C repeated three times with one-hour intervals. Approximately 1000 female flies were collected for analysis 24 h post induction and flash frozen in liquid N_2_. Microsomes were prepared according to standard procedures (Lee and Scott, 1989) and stored at -80 °C. Briefly, flies were homogenized using a Dounce homogenizer in 0.1 M Na/K-phosphate buffer, pH 7.4 containing 1 mM EDTA and DTT and 200 mM sucrose using a Fastprep (MP Biomedicals) and centrifuged for 10 min at 680*g* at 4 °C to pellet insoluble material. The supernatant was then centrifuged for 15 min at 10,000*g* at 4 °C and the supernatant of the second spin was centrifuged for 1 h at 100,000*g* at 4 °C, with the pellet subsequently resuspended in 0.1 M Na/K-phosphate buffer, pH 7.6 containing 1 mM EDTA and DTT and 10% glycerol using a Dounce tissue grinder. The protein content of samples was determined using Bradford reagent (Sigma) and bovine serum albumin (BSA) as a reference. Metabolism assays and UPLC-MS/MS analysis of thiacloprid metabolism was assayed by incubating microsomes prepared from transgenic lines (50 mg of protein/assay) or empty pUAST (control line) in 0.1 M potassium phosphate buffer with an NADPH-regenerating system (Promega; 1.3 mM NADP+, 3.3 mM glucose-6-phosphate, 3.3 mM MgCl2, 0.4 U ml-1 glucose-6- phosphate dehydrogenase) and substrate (50 μM) at 25 °C for 1 h. The total assay volume was 200 ml using three replicates for each data point. Microsomes incubated without NADPH served as a control. The assay was stopped by the addition of ice-cold acetonitrile (to 80% final concentration), centrifuged for 10 min at 3000*g* and the supernatant subsequently analysed by tandem mass spectrometry as described previously (Manjon et al., 2018). Substrate turnover was plotted versus controls using GraphPad Prism –v7 (Prism, 1994) (GraphPad Software, CA, USA).

## 3 Results and discussion

### 3.1 The Fly-Tox panel

The Fly-Tox panel comprises >30 fly lines containing P450 genes from 7 pest and 3 pollinator species (Table 1). Fifteen of these lines were created in previous studies and transgenic fly lines expressing three of these P450s have also been described previously but were recreated in this study to ensure all lines in the panel share the same genetic background. Finally, 12 lines are reported here for the first time. Further details and relevant citations on the previously published lines are provided in Table 1, and the lines are briefly described below. The panel is publicly available at the BDSC with BDSC stock numbers provided in Table 1.

### 3.2 CYP6ER1 variants and CYP6AY1

Four lines of the Fly-Tox panel express different ‘natural’ variants of the *N. lugens* P450 *CYP6ER1* which are expressed in brown plant hopper populations throughout Asia. These have not been previously published and include the two sequence variants primarily expressed in populations in Southeast Asia (*CYP6ER1vA*) and India (*CYP6ER1vB*). We demonstrate here that both fly lines expressing these P450s exhibit significant tolerance to the neonicotinoid imidacloprid compared to flies of the same genetic background without a transgene in insecticide bioassays (Table 1). Previous work has demonstrated that *D. melanogaster* expressing *CYP6ER1* are resistant to imidacloprid, thiamethoxam and buprofezin, however, the specific variant analysed in this prior study was not detailed (Wu et al., 2018). The natural *CYP6ER1* lines included in the panel are complemented by five ‘mutant’ variant lines which we published as part of a previous study (Zimmer et al., 2018). These comprise the ‘susceptible’ *CYP6ER1* variant (*CYP6ER1vL*) into which amino acid polymorphisms observed in ‘resistant’ *CYP6ER1* variants were introduced. The utility of these lines are detailed below. Finally we recreated a fly line expressing *CYP6AY1*, an alternative P450 that has been identified as overexpressed in imidacloprid resistant strains of *N. lugens* in China (Ding et al., 2013). In insecticide bioassays the line expressing this P450 exhibited significant but modest (1.7-fold) resistance to imidacloprid. However, when this P450 was expressed in *D. melanogaster* in a previous study the transgenic line showed no significant resistance to imidacloprid, thiamethoxam or buprofezin (Wu et al., 2018).

### 3.3 CYP6CY3/4

The Fly-Tox panel includes lines containing *CYP6CY3* and *CYP6CY4* from *M. persicae*. We have previously created a fly line expressing CYP6CY3 and demonstrated that this P450 confers tolerance to nicotine and the neonicotinoid insecticide clothianidin (Bass et al., 2013). We recently established that CYP6CY4, a second P450, is also overexpressed in nicotine resistant *M. persicae* (Singh et al., 2020) and demonstrate here that a fly line expressing this P450 also shows significant resistance to nicotine (Table 1).

### 3.4 CYP6BG1

In a previous study, a fly line expressing *CYP6BG1* of *P. xylostella* was shown to exhibit significant, albeit modest, tolerance to the diamide chlorantraniliprole in larval insecticide bioassays (Li et al., 2018). We have also previously created and performed bioassays with a transgenic fly line expressing this P450 which is included in the Fly-Tox panel. However, in our prior study this line showed no tolerance to chlorantraniliprole in the standard adult bioassay (see methods), indeed it was significantly more susceptible to this insecticide when compared to the control line lacking the transgene (Mallott et al., 2019). Thus, detection of a resistance phenotype using this line with this compound may require larval bioassays to be performed.

### 3.5 CYP6CM1 and CYP6BQ9

The Fly-Tox panel includes lines expressing two P450s, *CYP6CM1* of *B. tabaci* and *CYP6BQ9* of *T. castaneum*, that were among the first pest P450s to be expressed in *D. melanogaster (*Zhu et al., 2010; Daborn et al., 2012*)*. In adult bioassays, flies expressing *CYP6BQ9* exhibited significant tolerance to the pyrethroid deltamethrin and in larval bioassays, flies expressing *CYP6CM1* exhibited a modest (~2-fold) but significant level of resistance to the neonicotinoid insecticide imidacloprid (Karunker et al., 2008; Daborn et al., 2012).

### 3.6 CYP6BQ23 and CYP337B3

As detailed in the introduction, *CYP6BQ23* has been shown to confer resistance to the pyrethroids deltamethrin and *tau*-fluvalinate in *M. aeneus* and *CYP337B3* to fenvalerate and cypermethrin in *H. armigera* (Joußen et al., 2012; Rasool et al., 2014). Fly lines expressing each of these P450s were created and tested in adult bioassays with pyrethroids during the assembly of the Fly-Tox panel. However, the line expressing *CYP6BQ23* and the line expressing *CYP337B3* exhibited no tolerance to deltamethrin and fenvalerate respectively in adult bioassays. Therefore, additional optimisation of the bioassay protocol (such as testing the larval stage) may be required to observe a resistance phenotype using these lines.

### 3.7 Bee pollinator P450s

A total of 13 lines are included in the Fly-Tox Panel that contain P450s from three bee pollinator species. *CYP9Q1–3* from *A. mellifera*, *CYP9Q4–6* from *B. terrestris* and *CYP9BU1* and *CYP9BU2* from *O. bicornis* have been described previously (Manjon et al., 2018; Beadle et al., 2019), and when expressed in flies all but *CYP9BU2* confer tolerance to *N*-cyanoamidine neonicotinoids such as thiacloprid (Table 1). *CYP9R38* of *O. bicornis*, which has not been previously described, is much less closely related to the CYP9Q subfamily, confers no tolerance to this compound and therefore provides a useful additional control. Finally, new to this study are several fly lines which contain combinations of these P450s. These can be used to examine the effect of P450s on insecticide tolerance when combined (see below).

### 3.8 Applications of Fly-Tox panel

**Fig. 1:**
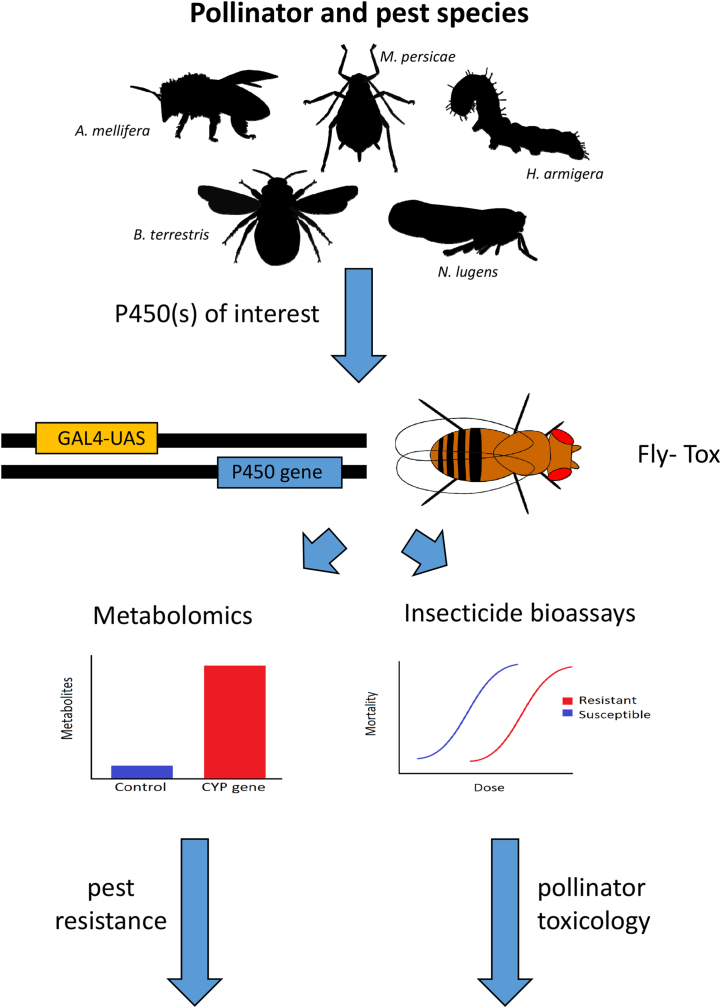
Schematic of Fly-Tox and its primary applications. Candidate genes from pest or pollinator species of interest are inserted into *D. melanogaster* using PhiC element integration. Expression of each gene is controlled using the GAL4-UAS system. The sensitivity of transgenic lines to insecticides can then be examined and/or insecticide breakdown products identified.

The Fly-Tox panel has numerous applications, below we outline some of the ways this resource can be used and summarise this information in Fig. 1.

#### 3.8.1 Determining cross-resistance/tolerance profiles

**Fig. 2:**
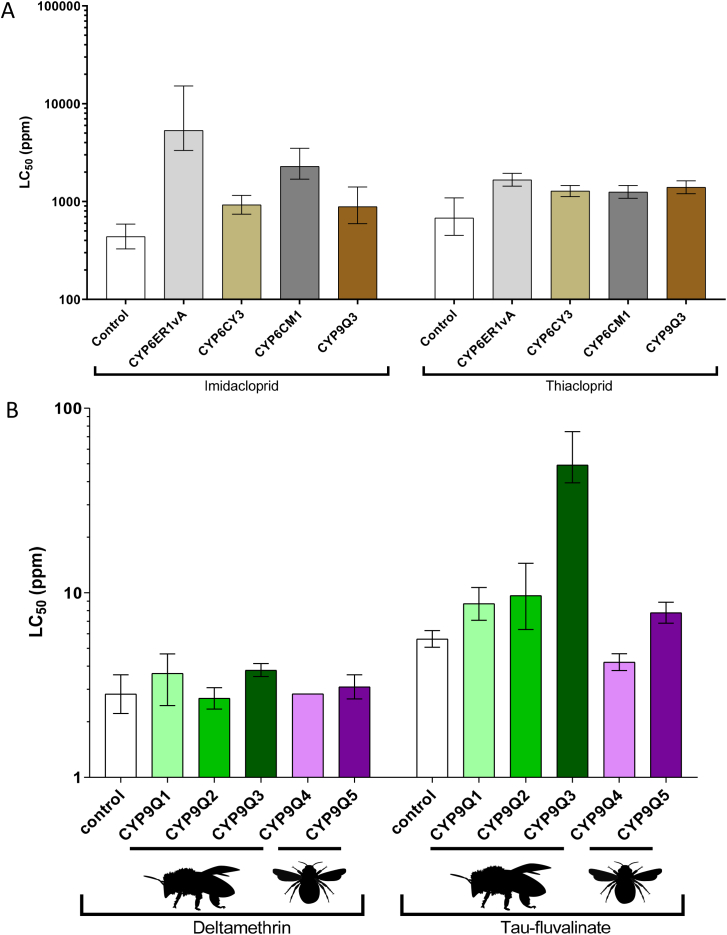
Using Fly-Tox to determine insecticide cross-resistance/tolerance profiles*.* A) Representative examples of screening fly lines expressing pest and pollinator P450s against the neonicotinoid insecticides imidacloprid and thiacloprid. Tested lines express *N. lugens* CYP6ER1, *M. persicae* CYP6CY3, *B. tabaci* CYP6CM2, and *A. mellifera* CYP9Q3. Results can be referenced against a control line carrying an empty transformation plasmid. B) Screen of two pyrethroid compounds (deltamethrin and *tau*-fluvalinate) against fly lines expressing P450s from two bee pollinator species. CYP9Q3 was previously implicated in conferring tolerance to *tau*-fluvalinate (Mao et al., 2011). Error bars on both graphs display 95% confidence limits.

One of the most obvious but important uses of the Fly-Tox panel is in predictive screens to examine the metabolic liability of new or existing insecticides. For example, once P450-mediated resistance to a certain insecticide is widespread in pest populations, alternative insecticides may be required to maintain control. In this case it is important to assess the efficacy of candidate replacements against field-relevant resistance. The Fly-Tox panel can be utilised to meet this need by using the relevant fly line to predict, at an early stage, if alternative chemistry is likely to be compromised by a specific resistance P450 (Fig. 2A). Likewise, any new compound can be tested during development or prior to release to determine if it is likely to be compromised by pre-existing resistance. Such a screening tool is especially valuable as predicting the cross-resistance profile of insect P450s in the absence of functional assays is notoriously difficult. In the same way, the pollinator P450s in the Fly-Tox panel can be used to determine if a new insecticidal compound under development is readily metabolised by bee P450s that provide protection against several existing insecticides (Fig. 2B). As outlined in the introduction, our previous work has demonstrated that these P450s can show marked differences in their capacity to metabolise different insecticides - even when these belong to the same class and share the same mode of action. If one or more of the bee P450-expressing fly lines exhibits tolerance to a compound in development, it provides an early indication that it may have low toxicity to bees, although further testing of the native pollinator would be required to unequivocally confirm this (see discussion).

#### 3.8.2 Identifying resistance-breaking inhibitors and avoiding negative pesticide-pesticide interactions

One established strategy for overcoming P450-mediated resistance is to use an inhibitor of this enzyme superfamily in combination with an insecticide. The inhibition of a key resistance P450 prior to, or in combination with, the application of an insecticide that is usually rapidly metabolised by that P450, can synergise insecticide action and restore susceptibility to a resistant pest population (Feyereisen, 2015). However, the capacity of P450s to be inhibited by well-known synergists such as piperonyl butoxide can vary. The Fly-Tox panel can be used to assess the effectiveness of candidate synergists to inhibit resistance-conferring P450 enzymes, in addition to their potential to restore insecticide susceptibility. The fly lines expressing pollinator P450s can be used in a similar way, but in this case to identify potential P450 inhibitors. This is particularly advantageous if the inhibitor may cause harm to pollinators if bees are exposed to them together with an insecticide that is metabolised by the P450. Such inhibitors can include other pesticides, for example, certain fungicides belonging to the azole class have shown to be potent inhibitors of bee P450s and can make insecticides that normally have low toxicity to bees much more toxic (Sgolastra et al., 2017). Identification of negative interactions in this way allows strategies to be put in place to mitigate risk, such as providing warnings on labels to avoid co-application/tank mixes etc.

#### 3.8.3 Understanding the structural and functional determinants of insecticide metabolism

**Fig. 3:**
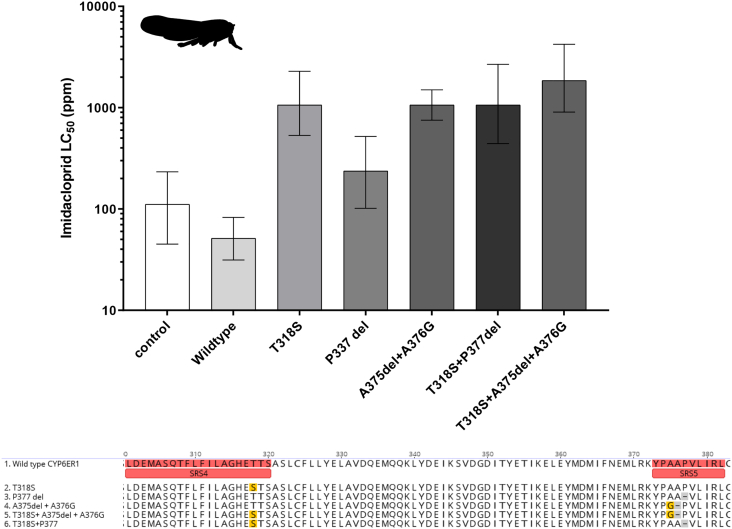
Using Fly-Tox to characterise the structural and functional determinants of insecticide metabolism. Sensitivity of fly lines expressing wildtype and mutant variants of *N. lugens* CYP6ER1. Amino acid substitutions identified in CYP6ER1 in imidacloprid resistant *N. lugens* strains were introduced singly or in combination into the wildtype (susceptible) CYP6ER1 variant and expressed in transgenic fly lines in order to understand their impact on sensitivity to this compound. These alterations occur in substrate recognition sites 4 and 5 as shown in the amino acid alignment (yellow shading substitution, grey shading deletion). Error bars represent 95% confidence limits. Data obtained from (Zimmer et al., 2018).

Our understanding of which amino acids in the active site of insect P450s are critical for binding and catalysis of insecticides is surprisingly limited. One route to gain insight into this topic is to characterise genetic variation in P450s in pest or pollinator populations and relate this to function (i.e. the ability of different P450 sequence variants to detoxify insecticide). We recently used this approach to show that only certain variants of the *N. lugens* P450 CYP6ER1 have the capacity to metabolise the insecticide imidacloprid (Zimmer et al., 2018). These are characterised by amino acid alterations in predicted substrate recognition sites. To identify which amino acid changes confer the ability to metabolise this insecticide we introduced substitutions/deletions, both individually and in combination, to the wild-type *CYP6ER1* variant and expressed them in an insect cell line. However, we observed marked differences in the expression of the different P450 variants in this system precluding robust comparative analysis. In contrast, expression of these P450s in transgenic flies, which are included in the Fly-Tox panel, allowed us to characterise the effect of these amino acid alterations on resistance and, in combination with homology modelling, understand how they alter the binding of imidacloprid in the active site (Fig. 3). This study demonstrates the utility of the *D. melanogaster* system for site-directed mutagenesis based functional analyses in order to understand the functional determinants of insecticide metabolism.

#### 3.8.4 *Understanding how the* spatiotemporal *expression of P450 affects its function*

As detailed in the introduction, a powerful aspect of the GAL4/UAS system is the flexibility it provides in terms of controlling the spatiotemporal expression of a gene of interest. To simply determine if a pest or pollinator P450 can confer tolerance to an insecticide, we and others commonly drive GAL4 expression using the Actin5C promoter which, when crossed to the UAS::P450 line, results in strong constitutive expression in all growing tissues. However, a wide range of alternative GAL4 driver lines are available that can be used to examine the effect of expressing an insecticide metabolising P450 in a specific tissue or under certain conditions (McGuire et al., 2004). Zhu et al., 2010. (Zhu et al., 2010) exploited this approach to express *CYP6BQ9* of *T. castaneum* in the central nervous system of flies and demonstrate that expression of this P450 in this tissue conferred resistance to deltamethrin. We also recently used this approach to direct the expression of *CYP9Q3* of *A. mellifera* to the Malpighian tubules and neuronal cells and showed that this was sufficient to provide protection to thiacloprid and acetamiprid (Manjon et al., 2018).

#### 3.8.5 Understanding P450 interactions

**Fig. 4:**
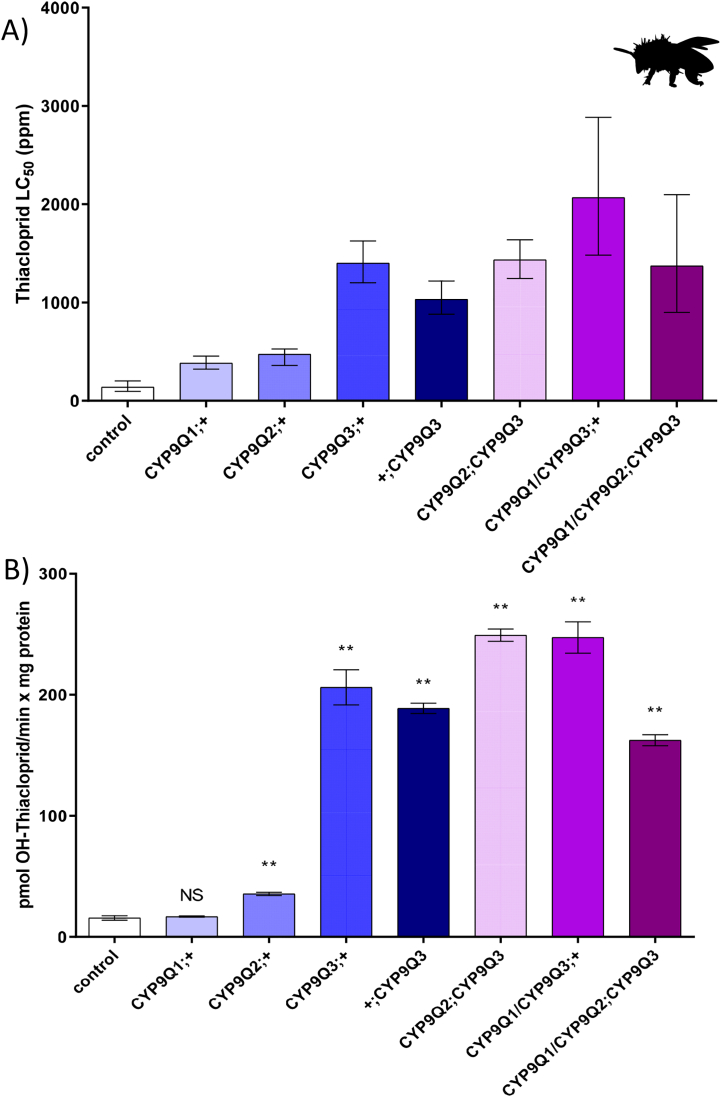
Using Fly-Tox to characterise P450 interactions and identify insecticide breakdown products. A) Sensitivity of fly lines expressing single or multiple P450s from *A. mellifera* to thiacloprid (lines containing multiple *A. mellifera* P450s were generated via either the integration of a single construct carrying multiple genes into chromosome 2 or multiple constructs carrying single genes into chromosomes 2 and 3). The expression of all genes was driven using Hsp-70-GAL4 promoter. Error bars display 95% confidence limits. B) Hydroxylation of thiacloprid to 4/5-Hydoxy thiacloprid by microsomes extracted from the same fly lines shown in panel A. Breakdown products identified previously (Manjon et al., 2018). Error bars display standard deviation of the mean. Statistical significance was calculated using One-way ANOVA with post-hoc Tukey HSD test (NS = no significance,**P < 0.01) relative to control group.

**Fig. 5:**
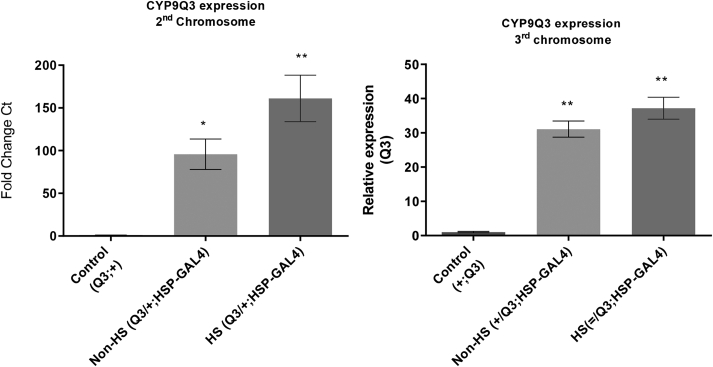
Positional effects of P450 transgene expression in ***D. melanogaster*.** Expression of CYP9Q3 driven by Hsp-70-GAL4 in transgenic flies when the transgene is integrated into attP40 on the second chromosome versus into attP86Fb on the third chromosome. Control is the parental line homozygous for the insertion of CYP9Q3. Gene expression was measured in flies carrying a single copy of both the CYP9Q3 construct and the Hsp70-GAL4 driver (progeny from the cross between CYP9Q3; + and +; Hsp70-GAL4 strains). Files were collected 48 h after heat-shock (H.S.). Error bars display 95% confidence limits. Statistical significance was calculated using One-way ANOVA with post-hoc Tukey HSD test (NS = no significance, *P < 0.05,**P < 0.01) relative to control group.

To our knowledge, to date, pest and pollinator P450s have been expressed in *D. melanogaster* in isolation. However, this system also allows P450s to be expressed together, in a single strain in order to assess their combined effect. Here we demonstrate the utility of this approach by creating and testing strains expressing multiple bee P450s, such as members of the CYP9Q subfamily of *A. mellifera*. Some of these strains presented an increased tolerance to thiacloprid when compared to strains expressing individual CYP9Q P450s (Fig. 4A, Table 1). We also highlight the importance of controlling for position effects when comparing these strains. The integration of CYP9Q3 at cytological position ZH-86Fb in chromosome 3 (*+;CYP9Q3*), for example, gives a lower level of expression compared to the integration of the same construct at cytological position 25C6 in chromosome 2 (*CYP9Q3;+*) (Fig. 5). As shown in Fig. 4A, *+;CYP9Q3* flies are also less tolerant to thiacloprid compared to *CYP9Q3;+.*

#### 3.8.6 Understanding insecticide metabolism

One perceived disadvantage of functional characterisation of pest and pollinator P450s using transgenic flies when compared to their characterisation in vitro, is the lack of information provided on the specific break-down products produced by a P450 of interest when incubated with an insecticide. While the transgenic fly system will never provide all the information generated by in vitro functional analysis, here we examined if it can be leveraged to provide insights into insecticide metabolites. We took the Fly-Tox lines expressing *CYP9Q1*, *CYP9Q2* or *CYP9Q3*, both singly and in combinations, extracted microsomal membranes (a source of total cytochrome P450 enzymes localized to the endoplasmic reticulum) and incubated these with the insecticide thiacloprid. We then used liquid chromatography–tandem mass spectrometry (LC–MS/MS) to quantify the amount of 5-hydroxy thiacloprid produced by the microsomal incubations of each line. We monitored for this specific metabolite as we have previously shown this is the primary product produced when recombinantly expressed CYP9Q1–3 are incubated with this insecticide (Manjon et al., 2018). We observed remarkable concordance between the production of 5-hydroxy thiacloprid in the three fly lines and both the formation of this metabolite by recombinant versions of these P450s and the level of tolerance they confer to this compound (Fig. 4B). These results show a clear correlation between the levels of metabolites produced by the transgenic strains, the level of CYP expression in those strains and the increased tolerance to the tested insecticide. In addition, these results also provide clear evidence that it is possible to utilise transgenic lines to understand how an insecticide is metabolised by a pollinator or pest P450.

## 4 Conclusion

As detailed in the introduction, the maintenance and testing of toxicologically relevant pest and pollinator species as part of pesticide research and development is challenging, expensive and in some cases only possible during certain months of the year (i.e. many bee species). In contrast, the Fly-Tox panel is inexpensive, easy to maintain and may be used year round. Furthermore, we anticipate that ourselves and others in the research community will, in future, add new lines to the current panel further enhancing its utility. However, we do not suggest that Fly-Tox should replace in vivo testing of native pests and pollinators. One of the main limitations of our panel, especially when testing novel compounds is their efficacy is *D. melanogaster* itself. Furthermore, if selectivity is an important driver, a compound class may work against the respective pest but not Drosophila (Douris et al., 2020). It is also important to acknowledge that the expression of a pest/pollinator P450 in *D. melanogaster* will never fully represent the cellular environment of the native species. These differences may affect the correct expression and/or function of the P450 of interest and therefore need to be considered when interpreting results and drawing conclusions. For example, P450s rely on co-factors for their catalytic activity, most notably cytochrome P450 reductase (CPR), which will also be contributed by the fly rather than the pest/pollinator (Murataliev et al., 1999). This may, in part, be the reason that the resistance levels conferred by pest P450s when expressed in *D. melanogaster* are frequently much lower than that observed in the native species. For example, expression of *CYP6CM1* in this system results in resistance levels of approximately 2-fold to imidacloprid compared to the 5–40 fold levels of resistance to this compound reported in Q and B biotypes of *B. tabaci* (Karunker et al., 2008). Despite these limitations, we conclude that the Fly-Tox panel has utility in a range of applications, as outlined above, including in early screens of lead compounds to establish if it is worthwhile conducting further, more costly, in depth testing.
