# Supplementary material for: Fly-Tox: A panel of transgenic flies expressing pest and pollinator cytochrome P450s
Source: Pestic Biochem Physiol. 2020 Oct;169:104674. doi: 10.1016/j.pestbp.2020.104674 (PMC7482442; doi:10.1016/j.pestbp.2020.104674)
Supplement: Supplementary material 1 [file mmc1.docx]

**Figure S1 A crossing scheme to generate fly strains expressing different combinations of CYP9Q1, CYP9Q2 and CYP9Q3. Abbreviations: Q1 = UAS-CYP9Q1; Q2 = UAS-CYP9Q2; Q3 = UAS-CYP9Q3; +/+ = wild-type chromosome; Sp/Cy; Sb/Tb-Hu = multi-balanced strain; P# = Progeny to be selected from # (cross).**

**First step - Balancing the chromosomes that will be recombined. The multi-balanced strain (Sp/Cy; Sb/Tb-Hu) was crossed to the five strains as shown below:**

#1-1 Q2/Q2; +/+ **X** Sp/Cy; Sb/Tb-Hu

P#1-1: **Q2/Sp; +/Tb-Hu**

#1-2 +/+; Q3/Q3 **X** Sp/Cy; Sb/Tb-Hu

P#1-2: **+/Cy; Q3/Sb**

#1-3 Q1/Q1; +/+ **X** Sp/Cy; Sb/Tb-Hu

P#1-3: **Q1/Sp; +/Tb-Hu**

#1-4 +/+; Hsp70-GAL4/ Hsp70-GAL4 **X** Sp/Cy; Sb/Tb-Hu

P#1-4: **+/Cy; Hsp70-GAL4/Sb**

**Second step - Selecting for chromosomal recombination. The presence/absence of balanced chromosomes allows the selections of flies carrying the genes of interest.**

#2-1 (P#1-1 X P#1-2) Q2/Sp; +/Tb-Hu **X** +/Cy; Q3/Sb

P#2-1: **Q2/Cy; Q3/Tb-Hu**

#2-3 (P#1-3 X P#1-5) Q1/Sp; +/Tb-Hu **X** +/Cy; Hsp70-GAL4/ Sb

P#2-3: **Q1/Cy; Hsp70-GAL4/Tb-Hu**

**Third step - Generating homozygous strains (stocks). Balanced chromosomes are removed and flies carrying two copies of each gene are selected.**

#3-1 (P#2-1 X P#2-1) Q2/Cy; Q3/Tb-Hu **X** Q2/Cy; Q3/Tb-Hu

P#3-1: **Q2/Q2; Q3/Q3** (Stock)

#3-2 (P#2-3 X P#2-3) Q1/Cy; Hsp70-GAL4/Tb-Hu **X** Q1/Cy; Hsp70-GAL4/Tb-Hu

P#3-2: **Q1/Q1; Hsp70-GAL4/Hsp70-GAL4** (Stock)

**Final step - Expressing multiple genes in single fly strains. Homozygous stocks are crossed and flies carrying the desired combinations of genes and drivers are selected.**

#4-5 (P#3-1 X P#3-2) +/+; Hsp70-GAL4/ Hsp70-GAL4 **X** Q2/Q2; Q3/Q3

P#4-5: **+/Q2; Hsp70-GAL4/Q3** (Flies expressing Q2 and Q3)

#4-6 (P#3-1 X P#3-2) Q1/Q1; Hsp70-GAL4 /Tb-Hu **X** Q2/Q2; +/+

P#4-6: **Q2/Q1; +/ Hsp70-GAL4** (Flies expressing Q1 and Q2)

#4-7 (P#3-1 X P#3-2) Q1/Q1; Hsp70-GAL4/Hsp70-GAL4 **X** +/+; Q3/Q3

P#4-7: **+/Q1; Q3/ Hsp70-GAL4** (Flies expressing Q1 and Q3)

#4-8 (P#3-1 X P#3-2) Q1/Q1; Hsp70-GAL4/Hsp70-GAL4 **X** Q2/Q2; Q3/Q3

P#4-8: **Q2/Q1; Q3/Hsp70-GAL4** (Flies expressing Q1, Q2 and Q3)

**Figure S2 Validation of expression of representative P450s in transgenic lines A) *B. terrestris* CYP9Q4 B) *B. terrestris* CYP9Q5 C) *B. terrestris* CYP9Q6 D) *A. mellifera* CYP9Q2. N=4 all error bars show 95% confidence limits.**

**
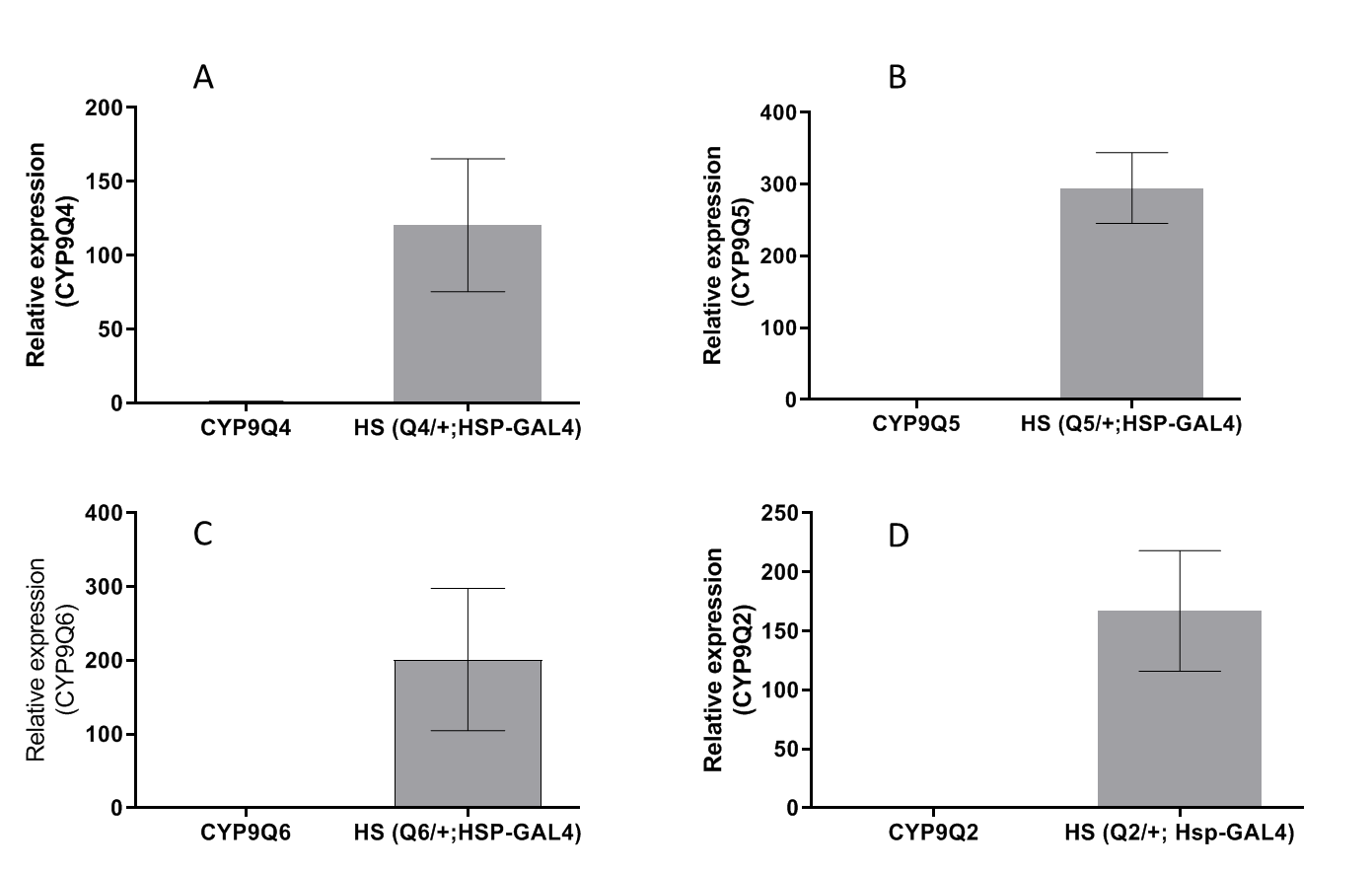
**

**Table S1 Genotypes of Drosophila lines submitted into fly-tox panel**

|  | **Genotypes** |
| --- | --- |
| CYP6CY3 | y w M(eGFP, vas-int, dmRFP)ZH-2A; P{ w[+mC]=UAS-Cyp6CY3}attp40 |
| CYP6CY4 | y w M(eGFP, vas-int, dmRFP)ZH-2A; P{ w[+mC]=UAS-Cyp6CY4}attp40 |
| CYP6ER1vL | y w M(eGFP, vas-int, dmRFP)ZH-2A; P{ w[+mC]=UAS-Cyp6ER1vL}attp40 |
| CYP6ER1vA | y w M(eGFP, vas-int, dmRFP)ZH-2A; P{ w[+mC]=UAS-Cyp6ER1vA}attp40 |
| CYP6ER1vB | y w M(eGFP, vas-int, dmRFP)ZH-2A; P{ w[+mC]=UAS-Cyp6ER1vB}attp40 |
| CYP6ER1vC | y w M(eGFP, vas-int, dmRFP)ZH-2A; P{ w[+mC]=UAS-Cyp6ER1vC}attp40 |
| CYP6ER1_P377del | y w M(eGFP, vas-int, dmRFP)ZH-2A; P{ w[+mC]=UAS-Cyp6ER1_P377del}attp40 |
| CYP6ER1_A375del+A376G | y w M(eGFP, vas-int, dmRFP)ZH-2A; P{ w[+mC]=UAS-Cyp6ER1_A375del+A376G}attp40 |
| CYP6ER1_T318S+P377del | y w M(eGFP, vas-int, dmRFP)ZH-2A; P{ w[+mC]=UAS-Cyp6ER1_T318S+P377del}attp40 |
| CYP6ER1_T318S+A375del+A376G | y w M(eGFP, vas-int, dmRFP)ZH-2A; P{ w[+mC]=UAS-Cyp6 ER1_T318S+A375del+A376G}attp40 |
| CYP6ER1_T318S | y w M(eGFP, vas-int, dmRFP)ZH-2A; P{ w[+mC]=UAS-Cyp6ER1_T318S}attp40 |
| CYP6AY1 | y w M(eGFP, vas-int, dmRFP)ZH-2A; P{ w[+mC]=UAS-Cyp6AY1}attp40 |
| CYP6BG1 | y w M(eGFP, vas-int, dmRFP)ZH-2A; P{ w[+mC]=UAS-Cyp6BG1}attp40 |
| CYP6CM1 | y w M(eGFP, vas-int, dmRFP)ZH-2A; P{ w[+mC]=UAS-Cyp6CM1}attp40 |
| CYP6BQ23 | y w M(eGFP, vas-int, dmRFP)ZH-2A; P{ w[+mC]=UAS-Cyp6BQ23}attp40 |
| CYP6BQ9 | y w M(eGFP, vas-int, dmRFP)ZH-2A; P{ w[+mC]=UAS-Cyp6BQ9}attp40 |
| CYP337B3 | y w M(eGFP, vas-int, dmRFP)ZH-2A; P{ w[+mC]=UAS-Cyp337B3}attp40 |
| CYP9Q1 | y w M(eGFP, vas-int, dmRFP)ZH-2A; P{ w[+mC]=UAS-Cyp9Q1}attp40 |
| CYP9Q2 | y w M(eGFP, vas-int, dmRFP)ZH-2A; P{ w[+mC]=UAS-Cyp9Q2}attp40 |
| CYP9Q3 | y w M(eGFP, vas-int, dmRFP)ZH-2A; P{ w[+mC]=UAS-Cyp9Q3}attp40 |
| CYP9Q4 | y w M(eGFP, vas-int, dmRFP)ZH-2A; P{ w[+mC]=UAS-Cyp9Q4}attp40 |
| CYP9Q5 | y w M(eGFP, vas-int, dmRFP)ZH-2A; P{ w[+mC]=UAS-Cyp9Q5}attp40 |
| CYP9Q6 | y w M(eGFP, vas-int, dmRFP)ZH-2A; P{ w[+mC]=UAS-Cyp9Q6}attp40 |
| CYP9BU1 | y w M(eGFP, vas-int, dmRFP)ZH-2A; P{ w[+mC]=UAS-Cyp9BU1}attp40 |
| CYP9BU2 | y w M(eGFP, vas-int, dmRFP)ZH-2A; P{ w[+mC]=UAS-Cyp9BU2}attp40 |
| CYP9R38 | y w M(eGFP, vas-int, dmRFP)ZH-2A; P{ w[+mC]=UAS-Cyp9R38}attp40 |
| **For combinations of different strains** |  |
| Q2/Q2; Q3/Q3 | y w M(eGFP, vas-int, dmRFP)ZH-2A; P{ w[+mC]=UAS-Cyp9Q2}attp40; M{3xP3-RFP.attP’ w[+mC]=UAS-Cyp9Q3}ZH-51C |
| Q1/Q1;HSP-GAL4/HSP-GAL4 | y w M(eGFP, vas-int, dmRFP)ZH-2A; P{ w[+mC]=UAS-Cyp9Q1}attp40; P{w[+mC]=GAL4-Hsp70.PB}89-2-1 |
| Q4/Q4;Q5/Q5 | y w M(eGFP, vas-int, dmRFP)ZH-2A; P{ w[+mC]=UAS-Cyp9Q4-Cyp9Q5}attp40 |
| Q1,2,3/Q1,2,3; +/+ (with spacers) | y w M(eGFP, vas-int, dmRFP)ZH-2A; P{ w[+mC]=UAS-Cyp9Q1-spacer-Cyp9Q2-spacer-Cyp9Q3}attp40 |
| Q4,5/Q4,5;+/+ (with spacers) | y w M(eGFP, vas-int, dmRFP)ZH-2A; P{ w[+mC]=UAS-Cyp9Q4-spacer-Cyp9Q5}attp40 |
| **Controls and GAL4 Driver lines** |  |
| Actin-GAL4 | y[1] w[*]; P{Act5C-GAL4-w}E1/CyO |
| Heat Shock-GAL4 | w[*]; P{w[+mC]=GAL4-Hsp70.PB}89-2-1 |
| Control empty pUAST chromosome 2 | y w M(eGFP, vas-int, dmRFP)ZH-2A; P{ w[+mC]=UAS}attp40 |
| Control chromosome 3 | y1 M{vas-int.Dm}ZH-2A w*; M{3xP3-RFP.attP'}ZH-51C |

**Table S2**

**Primer pairs used to validate insertions and expression levels**

| **Primer name** | **Sequence 5’-3’** | **Used in** |
| --- | --- | --- |
| **pUAST F** | TCACTGGAACTAGGCTAGCA | PCR and Sequence validation of transgenic flies |
| **pUAST R** | GGATCCAAGCTTGCATGCCTG | PCR and Sequence validation of transgenic flies |
| **RPS32 F** | GCGCTTGTTCGATCCGTAAC | qPCR housekeeping gene *D. melanogaster* |
| **RPS32 R** | GCCCAAGGGTATCGACAACA | qPCR housekeeping gene *D. melanogaster* |
| **Cyp9Q3 qPCR F** | GACCAGCAGCAAGATCAAGG | qPCR transgenic cyp9Q3 |
| **Cyp9Q3 qPCR R** | TCGTTGATGCTATCCACGGA | qPCR transgenic cyp9Q3 |
